# In Vitro Evaluation of the Influence of Substrate Mechanics on Matrix-Assisted Human Chondrocyte Transplantation

**DOI:** 10.3390/jfb11010005

**Published:** 2020-01-18

**Authors:** Yueh-Hsun Kevin Yang, Courtney R. Ogando, Gilda A. Barabino

**Affiliations:** Department of Biomedical Engineering, The City University of New York–the City College, New York, NY 10031, USA; cogando000@citymail.cuny.edu (C.R.O.); gbarabino@ccny.cuny.edu (G.A.B.)

**Keywords:** matrix-assisted chondrocyte transplantation, Young’s modulus, cartilage integration, cartilage explant model, human chondrocytes, agarose

## Abstract

Matrix-assisted chondrocyte transplantation (MACT) is of great interest for the treatment of patients with cartilage lesions. However, the roles of the matrix properties in modulating cartilage tissue integration during MACT recovery have not been fully understood. The objective of this study was to uncover the effects of substrate mechanics on the integration of implanted chondrocyte-laden hydrogels with native cartilage tissues. To this end, agarose hydrogels with Young’s moduli ranging from 0.49 kPa (0.5%, *w*/*v*) to 23.08 kPa (10%) were prepared and incorporated into an in vitro human cartilage explant model. The hydrogel-cartilage composites were cultivated for up to 12 weeks and harvested for evaluation via scanning electron microscopy, histology, and a push-through test. Our results demonstrated that integration strength at the hydrogel-cartilage interface in the 1.0% (0.93 kPa) and 2.5% (3.30 kPa) agarose groups significantly increased over time, whereas hydrogels with higher stiffness (>8.78 kPa) led to poor integration with articular cartilage. Extensive sprouting of extracellular matrix in the interfacial regions was only observed in the 0.5% to 2.5% agarose groups. Collectively, our findings suggest that while neocartilage development and its integration with native cartilage are modulated by substrate elasticity, an optimal Young’s modulus (3.30 kPa) possessed by agarose hydrogels is identified such that superior quality of tissue integration is achieved without compromising tissue properties of implanted constructs.

## 1. Introduction

Articular cartilage is a lubricant substrate that serves as a cushion between the bones of diarthrodial joints to absorb shock induced during joint movement. Multiple factors such as aging, disease, and abnormal loading conditions applied to the joints can cause cartilage degeneration. Among various forms, osteoarthritis (degeneration of cartilage and underlying bone) is the most common type of arthritis and often develops as people age [1]. Younger populations, however, can suffer from post-traumatic osteoarthritis mainly attributed to physical injury on the articular surface, such as sports, work, military injury, or car accident, which triggers the subsequent joint degenerative process [2]. Self-repair of articular cartilage is challenging mainly due to its avascular nature resulting in limited and inefficient nutrient supply, as well as reduced anabolic activities of mature chondrocytes residing within [3]. Several surgical treatments for cartilage defects are currently available, yet autologous chondrocyte implantation (ACI) is the only cell-based surgical therapy approved in the United States [4]. Alternatively, tissue engineering approaches which involve the use of a combination of cells, biomaterials, and bioactive molecules to grow functional tissue substitutes can offer a long-term solution to cartilage degeneration [5]. Inspired by tissue engineering strategies, a third-generation ACI product called matrix-induced autologous chondrocyte implantation or MACI^®^, where chondrocytes isolated from patients are first seeded on a collagen-based scaffold prior to implantation, has been developed and utilized clinically to overcome some issues observed in traditional ACI such as periosteal hypertrophy [6]. While traditional ACI can only be considered for patients with minor cartilage lesions, it becomes more effective in treating relatively large defects when combined with tissue engineering matrices. The approach is commonly known as matrix-assisted chondrocyte transplantation (MACT) and can be combined with both autologous and allogeneic cells.

Tissue grafts or tissue-engineered constructs need to integrate with patients’ native tissues to support their continued development after the implantation. Although it has been demonstrated that fibrocartilage, which is biochemically and mechanically inferior to healthy articular cartilage, usually forms at the implant–cartilage interface [7], several attempts to improve cartilage integration have been made. For example, treatment with N-benzyloxycabonyl-Val-Ala-Asp-fluoromethylketone [8] or an extracellular regulated kinase inhibitor [9], which suppresses cell apoptosis and production of pro-inflammatory cytokines, respectively, significantly strengthened the integrating cartilage tissues. Clinically, fibrin glue composed of fibrinogen is the standard material used to anchor cells and grafts to cartilage defect sites [10]. Although the use of fibrin glue enhances initial adhesion of the implant to native cartilage, stable integration has not been achieved due to the high degradation rate of fibrin glue, and thus it is considered as a delivery vehicle rather than a supporting substrate [11]. The presence of fibrin glue at the integration interface also creates a blocking boundary that potentially constrains cell migration and molecular exchange between the two compartments, which can accelerate the degeneration of the implant [7]. Therefore, methods for the optimization of naturally established cartilage integration must be sought to ensure long-term stability of the regenerating tissue.

In vivo, cells residing in different tissues and organs are exposed to diverse physical and mechanical environments. Experimentally, cells cultivated on or within a substrate have been shown to be able to recognize the changes in matrix stiffness through physical interactions, and thereby behave differently [12]. For example, Engler et al. showed that, in the absence of exogenous inductive molecules, mesenchymal stem cells exhibited neurogenic, myogenic, and osteogenic potential when cultured on the surface of collagen-modified polyacrylamide hydrogels with low, intermediate, and high elasticity, respectively [13]. Moreover, neural stem cells (NSCs) are highly sensitive to matrix elasticity such that NSC differentiation into three unique cell types (neurons, astrocytes and oligodendrocytes) is accomplished within a narrow mechanical interval ranging from 1 kPa to 7 kPa [14]. A more recent study suggests that polyethylene glycol hydrogels possessing relatively low Young’s moduli can facilitate production of proper extracellular matrix (ECM) components by the encapsulated human osteoarthritic chondrocytes [15]. Although the role of substrate stiffness in regulating differentiation and behavior of different cell types has been extensively explored [12,13,14,15,16,17], its influence on cartilage regeneration and integration in MACT therapy remains elusive.

This work aimed to uncover how substrate mechanics can contribute to cartilaginous tissue development and integration during MACT recovery. An in vitro disc-ring human cartilage explant model [18] was employed herein, which precisely controls, manipulates, and measures material properties and also excludes undesired variables such as immune responses that are commonly seen in in vivo studies. Among a variety of tissue engineering matrices, liquid-formed hydrogels are preferred over dry scaffolds due to their flexibility in shape and geometry which minimizes the initial gaps between implanted hydrogels and host tissues upon gelation. Nondegradable agarose that has been utilized in many cartilage tissue engineering applications [19,20] was selected as our model hydrogel system because of its inability to swell at a constant temperature after solidification [21], which eliminates potential interference in assessment of tissue integration strength attributed to volumetric expansion of the hydrogel. Our results suggest that while development of implanted neocartilage and its integration with native human cartilage are largely modulated by substrate stiffness, an optimal Young’s modulus (3.30 kPa) possessed by agarose hydrogels is identified such that superior quality of tissue integration is achieved without compromising biochemical and biomechanical properties of engineered cartilaginous constructs.

## 2. Results and Discussion

### 2.1. Agarose Hydrogels with Varied Mechanics

Thermoresponsive agarose hydrogels were fabricated from six different concentrations (0.5% to 10%, *w*/*v*), followed by solidification via temperature reduction. The selection of this range of agarose concentrations was mainly based on what has been commonly used in cartilage tissue engineering applications [19,20,22,23]. Experimentally, the Young’s moduli were 0.49 kPa, 0.93 kPa, 3.30 kPa, 8.78 kPa, 14.60 kPa, and 23.08 kPa in the 0.5%, 1.0%, 2.5%, 5.0%. 7.5%, and 10% agarose hydrogels, respectively, while the shear moduli increased from 24.70 kPa to 902.28 kPa (Table 1). The water content within each gel type decreased with increasing agarose concentration from 99.60% in the 0.5% hydrogels to 92.07% in the 10% samples. Similarly, the mass swelling ratio, an indicator of the degree of void space present in a hydrogel, reduced significantly from 236.50 ± 2.30 in the 0.5% agarose group to 8.73 ± 0.39 in the 10% group. Taken together, hydrogels with gradient mechanics were successfully prepared while their other physical properties also changed accordingly. Although the Young’s modulus values measured, herein, are comparable to those reported by Benkherourou et al. [24], divergent outcomes have also been documented which can be attributed to multiple factors including, but not limited to, the type of agarose, agarose viscosity, and mechanical testing (for example, confined versus unconfined compression and local versus bulk property) [19,22,25,26,27]. Overall, our data suggest that agarose contents largely contribute to the initial mechanical strength possessed by hydrogels and limited unfilled space exists in the network of hydrogels with higher agarose concentrations.

### 2.2. Establishment of InVitro Disc-Ring Hydrogel-Cartilage Explant Model that Mimics MACT

Human whole femurs were used for extraction of intact articular cartilage explants. The tissues were devitalized through freeze–thaw cycles to eliminate potential cellular activities and preserve ECM structure [28]. Cartilage explants were then shaped into discs whose mechanical and biochemical properties were quantified, as shown in Table 2. Briefly, the created tissue discs (∅8 mm) were 1.85 mm thick on average and had an equilibrium modulus of 871.24 kPa, a glycosaminoglycan (GAG) content of 13.93% of wet weight, a collagen content of 20.59% of wet weight, and a water content of 69.48%.

**Figure 1:**
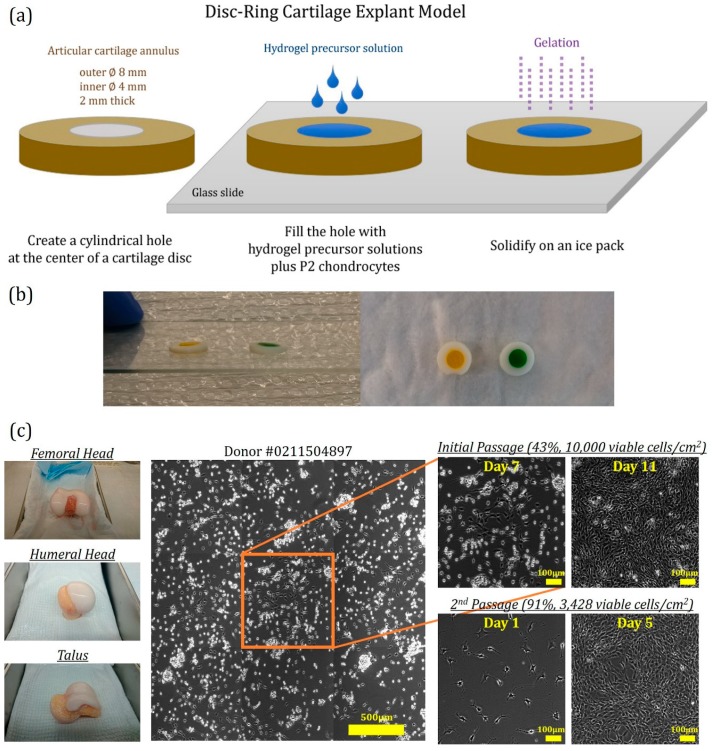
(**a**) Assembly of cartilage explant model. The central hole of a cartilage disc was filled with agarose precursor solutions containing 40 × 10^6^ human chondrocytes/mL, and gelation was carried out on an ice pack for 1 to 3 min. (**b**) Representative photos of hydrogel-cartilage composites. The cartilage explant model was loaded with colored agarose gels prepared from either 0.5% (yellow) or 10% (green) precursor solutions. (**c**) Isolation of human articular chondrocytes. Primary chondrocytes were extracted from human osteochondral grafts of multiple donors including femoral head, humeral head, and talus and were passaged at a high seeding density (10^4^ cells/cm^2^) for up to 12 days to remove nonviable cells. Prior to each experiment, the second passage (P2) was performed at a lower seeding density (3428 cells/cm^2^) for 5 days to expand the chondrocyte population. Due to availability and viability of osteochondral grafts, P2 cells from different osteochondral origins were pooled together and used in the experiments. Scale bars: 500 µm or 100 µm.

**Figure 2:**
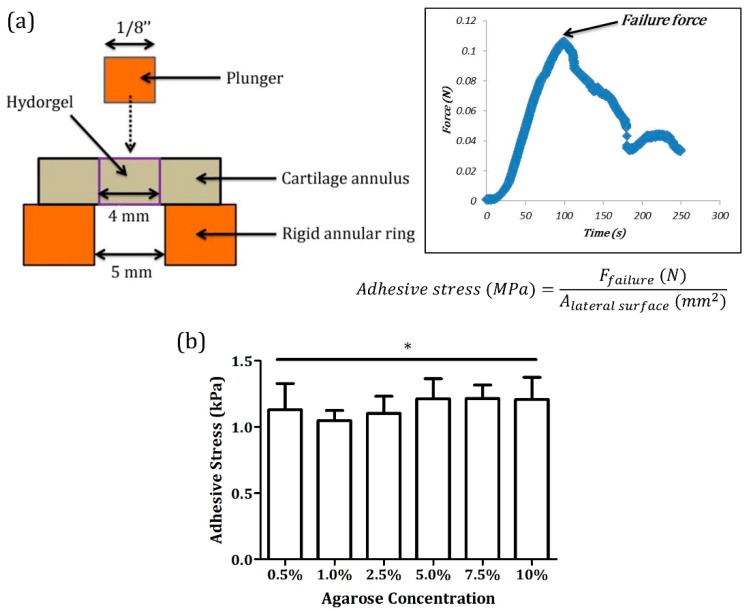
(**a**) Push-through test. Quality of cartilage tissue integration was determined by measuring integration or adhesive strength at the hydrogel–cartilage interface using a push-through test. During the examination, the implant was gradually pushed out by a plunger at a constant rate to obtain the failure force that was used to calculate adhesive stress. (**b**) Adhesive strength of hydrogel-cartilage composites on Day 1. No significant variations in adhesive stress were detected in Day 1 samples across different groups, implying that agarose swelling did not contribute to or influence cartilage tissue integration. * Non-significance, *p* = 0.3458, and n = 5.

A ∅4 mm central hole was subsequently created in each of the explant discs to obtain cartilage annuli and the hole was filled with one of the hydrogel precursor solutions (Figure 1a,b). Human articular chondrocytes (Figure 1c) isolated from non-diseased osteochondral grafts were mixed with hydrogel precursor solutions before being loaded onto the cartilage explant model. After the hydrogels solidified, the disc-ring hydrogel-cartilage composites were cultured at 37 °C, 5% CO_2_ for up to 12 weeks. A push-through test (Figure 2a) performed on Day 1 of the cultivation revealed that the initial affinity or adhesive strength between the implanted hydrogel and the surrounding cartilage annulus did not alter with agarose concentration and was around 1.15 kPa in all the groups (* *p* > 0.05, Figure 2b), suggesting that volumetric swelling degree of agarose hydrogels has minimal effects on cartilage tissue integration potentially due to its inability to expand in size when exposed in an aqueous solution [21]. Interestingly, the composites loaded with chondrocyte-encapsulated 10% agarose hydrogels dissociated after five to seven days in culture, and thus were excluded from the subsequent experiments.

### 2.3. Integration of Chondrocyte-Laden Agarose Hydrogels with Human Articular Cartilage

**Figure 3:**
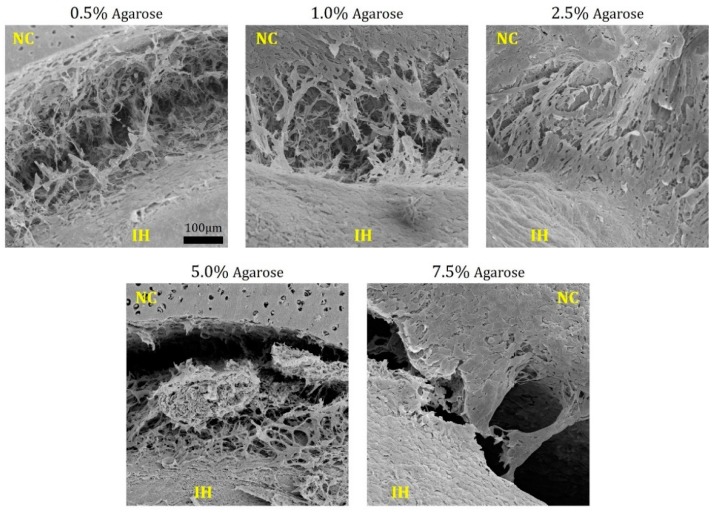
Scanning electron microscopy of hydrogel-cartilage composites at week 12. Extensive matrix sprouting was observed on the composite surface in the 0.5% to 2.5% agarose groups. NC and IH refer to native cartilage tissues and implanted hydrogels, respectively. The images were captured at 220× magnification. Scale bar: 100 µm.

Matrix sprouting at the implant–native tissue interface is a key contributing factor to integration of the two compartments [7]. A different extent of matrix sprouting was identified on or close to the surface of each type of hydrogel-cartilage composites at week 12, as shown by scanning electron microscopy (SEM, Figure 3). Specifically, implants composed of 0.5%, 1.0%, and 2.5% agarose resulted in extensive matrix sprouting, whereas wide gaps still existed in the composites filled with 5.0% and 7.5% hydrogels. Of note, most of the hydrogel-cartilage composites were covered with a thin layer of fibrous tissues. The formation of this fibrous outgrowth can be attributed to the direct contact of the composite surface with fetal bovine serum (FBS) present in culture media which contains biomolecules such as transforming growth factor-β (TGF-β) [29] and platelet-derived growth factor (PDGF) [30,31] that actively facilitate fibrotic mechanisms.

**Figure 4:**
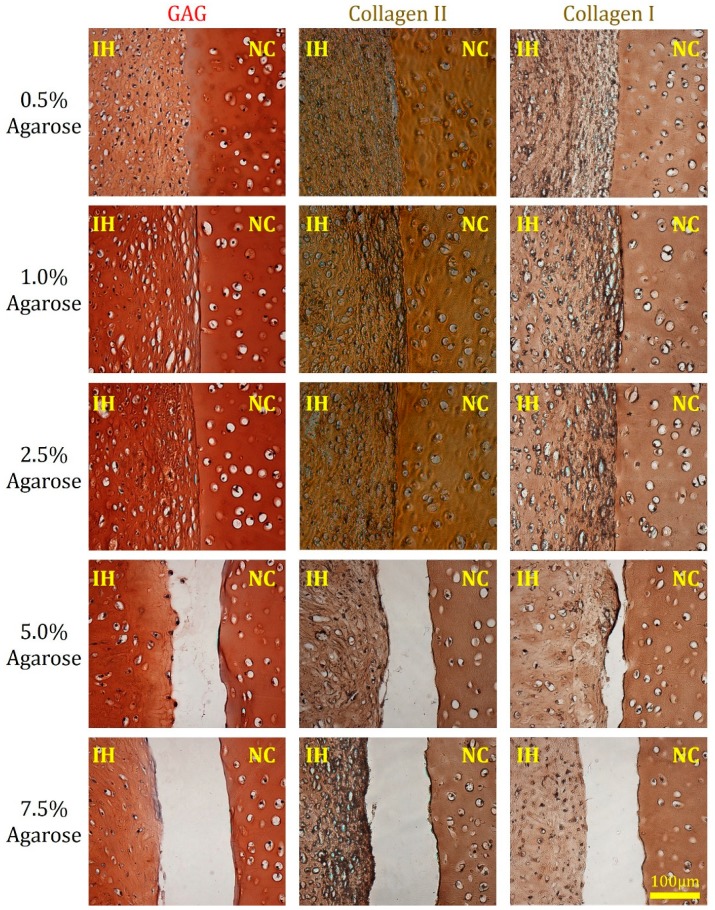
Histological and immunohistochemical staining of hydrogel-cartilage composites at week 12. Superior cartilage integration was observed in the composites loaded with 0.5%, 1.0%, and 2.5% agarose hydrogels. GAG molecules (left column) are stained red while type II collagen (middle column), and type I collagen (right column) are stained brown. NC and IH refer to native cartilage tissues and implanted hydrogels, respectively. Scale bar: 100 µm.

Inner portions of the hydrogel-cartilage composites were evaluated by histology and immunohistochemistry (Figure 4). While chondrocyte-laden agarose implants derived from all the groups expressed cartilage-specific ECM components, i.e., GAG and type II collagen, at week 12, the quality of their integration with native cartilage was quite divergent. In the 0.5%, 1.0%, and 2.5% agarose groups, merging areas at the hydrogel-cartilage boundary were widely observed. Conversely, most of the interfacial regions remained unfilled in the 5.0% and 7.5% agarose samples. Similar to many in vivo [32,33,34] and in vitro [18,35,36] experiments, deposition of type I collagen was also detected around the boundary between implanted hydrogels and native cartilage tissues, indicating frequent formation of fibrocartilage at defect or integration sites. Although it has been shown that articular chondrocytes can lose their phenotype and behave like fibroblasts after being passaged in a two-dimensional environment [37], our collagen staining results, i.e., stronger staining intensity of collagen II than collagen I in all the implants, suggest that chondrocyte phenotype was properly preserved after two passages in the presence of a unique growth factor cocktail composed of TGF-β1, PDGF-bb, and basic fibroblast growth factor (b-FGF). Such growth factor combination has been shown to better maintain proliferative and chondrogenic potential of passaged human chondrocytes [38]. On the basis of the qualitative assessment, amongst all the conditions, 2.5% agarose hydrogels encapsulated with human chondrocytes achieved superior tissue integration both on the surface (Figure 3) and along the full depth of the composite (Figure 4).

**Figure 5:**
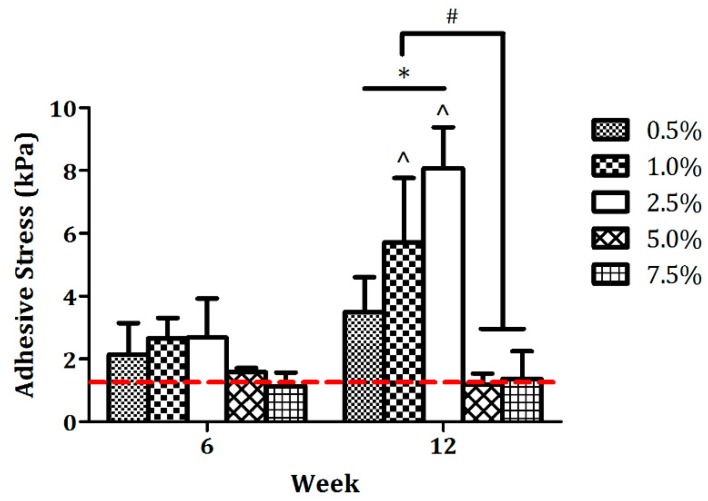
Integration strength of hydrogel-cartilage composites. No statistical significance was detected between any two groups at week 6. Adhesive stress increased substantially from week 6 to week 12 in both 1.0% and 2.5% groups, whereas the 5.0% and 7.5% values remained unchanged throughout the entire 12 weeks of composite culture. The red dashed line indicates the baseline of adhesive stress, i.e., initial affinity determined on Day 1 of the integration process, which was averaged from Figure 2b. *,# Significance between the groups, * *p* < 0.05, and # *p* < 0.001. ^ Significance versus the corresponding 6-week values, *p* < 0.05 for the 1.0% agarose group, *p* < 0.001 for the 2.5% agarose group, and n = 5.

Integration strength, also referred to as adhesive stress at the hydrogel–cartilage interface, was quantified at weeks six and 12 (Figure 5), and the data are in line with the microscopic results. Specifically, the average adhesive stresses in the 0.5%, 1.0%, and 2.5% groups were slightly higher than those of the other two groups at week six, although there was no statistical significance across different conditions. At week 12, integration strength in the 5.0% and 7.5% samples remained extremely low which was similar to the initial affinity level measured on Day 1 of the composite culture, whereas the 1.0% and 2.5% values continued to increase over time and were significantly higher than the corresponding six-week counterparts (^ *p* < 0.05) with a peak value of 8.07 ± 1.31 kPa detected in the 2.5% group. Taken together, our findings suggest that 2.5% agarose hydrogels offer an optimal mechanical environment for in situ integration of human chondrocyte-laden constructs with native cartilage.

It is believed that the quality of naturally established tissue integration relies on a variety of factors including, but not limited to, the age of the patient [39], the nature of cells [35] and substrates [40] used for implantation, the maturity of the implant [41], and the structure and composition of the implant and adjacent tissues [42]. In a study conducted by Obradovic et al., despite inferior mechanical properties, immature neocartilage integrated better with native tissues as compared with functional cartilaginous constructs, suggesting that a highly dense matrix network can hinder cartilage tissue integration [41]. This can be one explanation for the poor integration observed in our 5.0% and 7.5% agarose samples. Rationally, manipulation of hydrogel mechanics in most of the currently available gel fabrication approaches is achieved by adjusting the concentration of its base polymeric material or monomer [43,44,45,46]. Therefore, hydrogels with initial high stiffness can impede their integration with native tissues mainly because such tissue replacements are overloaded with gel monomers, i.e., agarose in this work, and therefore the permeability of constructs is extremely low, leading to inefficient nutrient transport and further limited tissue development and regeneration [3,47,48].

At the cellular level, cell migration also plays a key role in tissue integration. It is suggested that when implanted cells or host cells migrate across the implant-native tissue boundary, they simultaneously produce and deliver ECM components to the interfacial area, which eventually facilitates gap closing at the defect site [7]. We reasonably speculate that the 0.5% agarose hydrogels contained much void space within the network, as shown by the measurement of mass swelling ratio (Table 1), such that the encapsulated chondrocytes had to first fill those gaps with synthesized ECM before they could migrate toward the integration site, resulting in compromised adhesive stress in comparison with the 1.0% and 2.5% groups. However, cell motility within different types of hydrogels requires further thorough investigation.

### 2.4. Impact of Cartilage Annuli on Neocartilage Development

**Figure 6:**
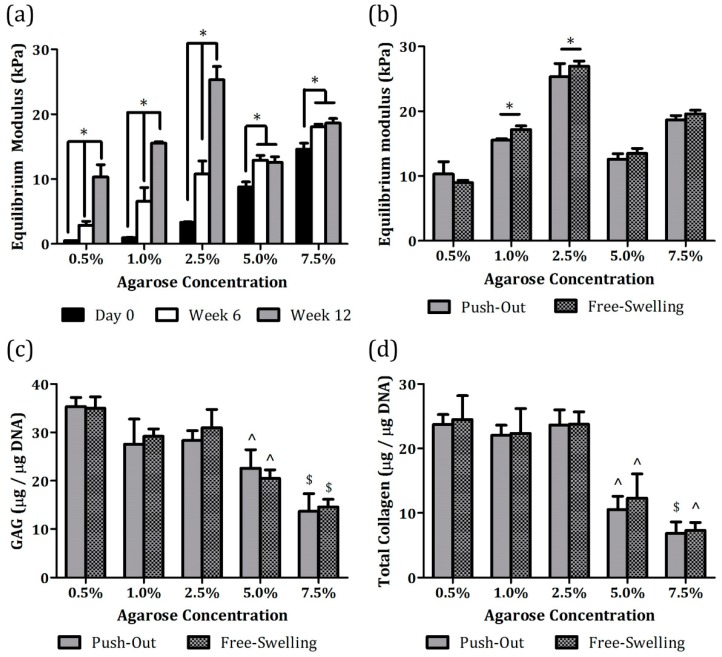
Tissue properties of chondrocyte-laden agarose hydrogels cultivated with or without a cartilage annulus: (**a**) Equilibrium moduli of implants were quantified at weeks 6 and 12 immediately after the push-through test, and the values were compared to the initial mechanical strength of agarose hydrogels on day 0, (**b**) equilibrium moduli, (**c**) GAG, and (**d**) collagen synthetic activities were compared between pushed-out and free-swelling cartilaginous constructs at week 12. * Significance between the groups, ^ significance versus the corresponding 0.5% to 2.5% agarose values, ^$^ significance versus the corresponding 0.5% to 5.0% agarose values, *p* < 0.05, and n = 5.

After the push-through test, the pushed-out implants were collected for assessment of tissue properties. Mechanically, equilibrium moduli of the 0.5%, 1.0%, and 2.5% cartilaginous samples continuously increased over time (* *p* < 0.05, Figure 6a) and by week 12, they were at least seven-fold stronger than the initial hydrogels (0.5%, 20.1 folds; 1.0%, 15.6 folds; and 2.5%, 7.7 folds). Conversely, despite some extent of improvement, the 5.0% and 7.5% values remained similar after six weeks in culture and the increase was less than 45% as compared with the corresponding levels on Day 0 (5.0%, 43.3% and 7.5%, 27.6%). This phenomenon can be attributed to reduced ECM synthetic activities in the 5.0% and 7.5% constructs as compared with the other groups (^^,$^ *p* < 0.05, Figure 6c,d). It also confirms that a highly dense hydrogel network tends to suppress anabolic abilities of the encapsulated cells. Overall, the strongest equilibrium modulus was accomplished by the chondrocyte-laden 2.5% agarose hydrogels at week 12, whose value was around 25.31 ± 2.04 kPa.

Pushed-out implants were also compared with free-swelling constructs, i.e., chondrocyte-encapsulated agarose hydrogels cultivated without a cartilage annulus, at week 12. Surprisingly, both types of specimens had comparable equilibrium moduli (Figure 6b), GAG (Figure 6c), and collagen (Figure 6d) synthetic activities in all the groups. Although a statistically significant difference in equilibrium modulus was determined between pushed-out and free-swelling values in the 1.0% and 2.5% agarose groups (* *p* < 0.05, Figure 6b), the variations were within 10% (1.0%, 9.5% and 2.5%, 6.1%). Moreover, chondrocytes encapsulated in the 0.5%, 1.0%, and 2.5% agarose hydrogels produced collagen molecules at a consistent rate regardless of culture format (Figure 6d) whereas those in the 0.5% agarose constructs had slightly stronger GAG anabolic potential (Figure 6c). Collectively, our results demonstrate that the presence of cartilage annuli did not really disturb development of implanted constructs as compared with the free-swelling counterparts. The use of devitalized cartilage in the establishment of the in vitro explant model could account for this outcome. Several studies that employed viable cartilage annuli from juvenile animals revealed their potential roles as a nutrient sink due to dense ECM and viable cells residing within, possibly delaying implant development and tissue integration [36,42]. It is also suggested that devitalized tissues can better emulate in vivo pathological conditions while preserving ECM components and architecture [28,36]. In addition, a more realistic problem is the difficulty of acquiring completely viable human cartilage tissues for research purpose.

## 3. Materials and Methods

### 3.1. Materials

Unless specified otherwise, reagents were purchased from Thermo Fisher Scientific (Waltham, MA, USA) or Sigma (St. Louis, MO, USA).

### 3.2. Characterization of Agarose Hydrogels

Hydrogel precursor solutions were prepared by dissolving agarose (type VII, Thermo Fisher Scientific, Waltham, MA, USA) in ultrapure water at 0.5%, 1.0%, 2.5%, 5.0%, 7.5%, and 10%, *w*/*v*. The solutions were casted between two parallel glass plates separated by a ~2 mm thick spacer and allowed to solidify on an ice pack for 1 to 3 min. To determine water content and mass swelling ratio, hydrogels were weighed after incubation with culture media at 37 °C, 5% CO_2_ overnight (wet weight). The gels were then completely dried in a freeze dryer (Labconco, Kansas City, MO, USA) and the dry weight was obtained. Water content was determined by normalizing the water weight to the wet weight, and mass swelling degree was shown as a ratio of the wet weight to the dry weight. Hydrogel stiffness was measured using a TA.XTplus texture analyzer (Stable Micro Systems, Godalming, UK) with a 1/8 inch spherical probe which compresses the samples at a rate of 10 µm/s until 10% strain is reached. Force (*F*), depth of indentions (*δ*), and strain (*ε*) data were recorded to calculate the contact radius (*a*), indentation stress (*σ*), Young’s modulus (*E*), and shear modulus (*G*) using the following equations [49]:

$$a\;=\;R^{1/2}\delta^{1/2}$$

$$\sigma \;=\;\frac{F}{\pi a^{2}}$$

$$E\;=\;\frac{3\pi \left(1\;-\;\nu^{2}\right)\sigma }{20\varepsilon }$$

$$G\;=\;\frac{3F}{16af_{p}\left(\frac{a}{h}\right)};\;f_{p}\left(\frac{a}{h}\right)\;=\;\frac{2.36{\left(\frac{a}{h}\right)}^{2}\;+\;0.82\left(\frac{a}{h}\right)\;+\;0.46}{\frac{a}{h}\;+\;0.46}$$

where *R* is the radius of the probe (1/8 inch), *ν* is Poisson’s ratio (0.5) [25], and *h* is the sample height.

### 3.3. Isolation and Expansion of Human Articular Chondrocytes

Human articular cartilage was harvested from expired, but non-diseased osteochondral grafts extracted from corpses (cat. # 460524, # 460525, Musculoskeletal Transplant Foundation, Edison, NJ, USA). Chondrocytes were then isolated through digestion of minced cartilage with collagenase type II followed by a series of centrifugation and wash steps, as previously described [50]. Prior to encapsulation in hydrogels, viable primary chondrocytes were passaged twice in the basal medium (high glucose Dulbecco`s modified Eagle media [hgDMEM], 1% penicillin-streptomycin-fungizone [PSF], 1% insulin-transferrin-selenium [ITS], 1% non-essential amino acid [NEAA], 0.9 mM sodium pyruvate, 3.72 mg/mL sodium bicarbonate, 40 µg/mL ascorbic acid and 40 µg/mL L-proline) supplemented with 2% FBS, 100 nM dexamethasone, 1 ng/mL TGF-β1, 10 ng/mL PDGF-bb and 5 ng/mL b-FGF (Figure 1c). To minimize inter-donor variation, passage 2 (P2) cells from five donors (Table 3) were pooled together and used in the experiments.

### 3.4. Assembly and Culture of Disc-Ring Cartilage Explant Model

Cartilage explants were extracted from human whole femurs collected from three donors (Table 3) and were shaped into discs (Ø8 mm × 2 mm thick) by aseptically removing both superficial and deep zone cartilage tissues. A Ø4 mm central core was then punched out of each disc and the hole was filled with one of the hydrogel precursor solutions containing 40 million viable P2 chondrocytes per milliliter of gel precursor solutions followed by 1 to 3 min gelation on an ice pack (Figure 1a,b). Disc-ring or hydrogel-cartilage composites were individually cultivated in 12-well plates with the basal medium containing 10% FBS. After 6 to 12 weeks in culture, the composites were harvested and evaluated via histology, SEM, and a push-through test. For comparison, free-swelling cell-laden agarose hydrogels without an outer cartilage annulus were cultured in parallel under identical conditions.

### 3.5. Push-Through Test

Integration strength or adhesive stress was determined by a push-through test [18] (Figure 2a) performed on a TA.XTplus texture analyzer in which the hydrogel implant of a composite was gradually pushed out by a 1/8 inch plunger at a rate of 10 µm/s, while the composite is placed on a custom-designed rigid annular ring with a 5 mm central hole. The force at ultimate failure was normalized to the lateral area of the inner disc to calculate adhesive stress. In addition, the push-through test was performed on day-one samples to assess integration strength and confirm that the volumetric swelling effects of agarose hydrogels were negligible.

### 3.6. Scanning Electron Microscopy

Tissue composites harvested at week 12 were fixed in 2% glutaraldehyde solution for 48 h at 4 °C and then dehydrated in a graded ethanol series (30%, 50%, 75%, 95%, and 100%). Following the critical drying process, the dried samples were mounted onto aluminum stubs (Electron Microscopy Sciences, Hatfield, PA, USA) and coated with a thin layer of carbon (Desk II, Denton Vacuum, Moorestown, NJ, USA). The coated specimens were viewed using a scanning electron microscope (Supra 55, Zeiss, Oberkochen, Germany).

### 3.7. Histology and Immunohistochemistry

Hydrogel-cartilage composites were fixed in 10% formalin and, then, embedded in paraffin. The paraffin blocks were sectioned in 5 μm thick slices which were later deparaffinized and stained for different ECM components. GAG molecules were visualized by staining deparaffinized sections with safranin-O. Type I and II collagens were stained using an immunohistochemical method. Briefly, slices were first immersed in a series of solutions including citrate buffer (99 °C), hydrogen peroxide (0.3%), and goat serum (1%) based blocking buffer. The samples were then incubated overnight with rabbit anti-human collagen I or II antibodies (Abcam, Cambridge, MA, USA) and sequentially with biotinylated goat anti-rabbit IgG (Abcam) and streptavidin-conjugated horseradish-peroxidase complex (Vector Labs, Burlingame, CA, USA), followed by the addition of diaminobenzidine chromogen reagent. The negative staining control was established by incubating sections derived from the 2.5% agarose constructs with normal rabbit serum in lieu of primary antibodies, and no nonspecific staining was observed (images are available upon request). Color images were captured under a light microscope (Nikon Eclipse Ti, Tokyo, Japan).

### 3.8. Mechanical and Biochemical Analyses of Neocartilage

Pushed-out and free-swelling cartilaginous constructs were assessed for equilibrium moduli using an unconfined compression test [51] (ElectroForce 3100, TA Instrument, New Castle, Germany). Each sample was first compressed with a preload of 0.01 N. A stress relaxation test was then carried out at strains of 5%, 10%, 15%, 20%, and 25% in which sufficient time was given until equilibrium was achieved after each applied strain rate. Equilibrium moduli were calculated from the slope of the plot of equilibrium forces normalized to the cross-sectional area of the construct versus the strains.

Tissue-engineered constructs were weighed (wet weight), frozen, lyophilized, and digested with papain enzyme for 17 h at 60 °C followed by biochemical evaluation for DNA, GAG, and total collagen. Specifically, DNA was quantified using a PicoGreen dsDNA kit employing a fluorescence spectrometer [52]. The sulfated GAG content retained inside the construct was assessed spectrophotometrically at 525 nm using a 1,9-dimethylmethylene blue dye-binding assay [53]. Chondroitin sulfate was used to create a standard curve and a ratio of chondroitin sulfate to GAG of 1 was assumed. The total collagen content was determined using an orthohydroxyproline (OHP) colorimetric assay, assuming a 1 to 10 OHP-to-collagen concentration ratio [54]. The concentration of hydroxyproline was measured spectrophotometrically at 550 nm after acid hydrolysis and reaction with chloramine-T and p-dimethylaminobenzaldehyde. The GAG and collagen levels are presented in values normalized to the corresponding wet weight or DNA content of the construct.

### 3.9. Statistical Analyses

Statistical data show means ± one standard deviation. Statistical analyses were performed by one-way or two-way analysis of variance (ANOVA) in conjunction with the Bonferroni’s post-hoc test for multiple comparisons, and a *p*-value of less than 0.05 was considered as statistical significance.

## 4. Conclusions

To the best of our knowledge, this work, for the first time, provides a preliminary understanding of how substrate stiffness influences cartilage tissue integration during MACT recovery. The use of the in vitro cartilage explant model simplifies the complex in vivo conditions and experimental variables so that material properties of hydrogels can be exclusively studied. While the designed experiments were mainly built upon matrix elasticity (0.49 to 23.08 kPa, Table 1), one should keep in mind that other physical properties of hydrogels also change accordingly with mechanics. Nevertheless, it is difficult to decouple the interaction between those parameters at this point. In addition, volumetric expansion of implanted hydrogels, herein, was considered to be consistent across different groups due to the fact that agarose gels do not tend to swell once solidified. Thus, its effects on tissue integration remain unknown and can be evaluated using swollen hydrogels such as polyethylene glycol [43], polyacrylamide [44], and collagen gels [55]. The strongest integration strength achieved in this study was around 8 kPa within a 12 week integration process which is still much lower than the native tissue level. Although this affinity could be further improved by extending the length of the culture period [56], it substantiates the need to consider incorporation or modification of adhesive properties of gel monomers when new hydrogel systems are being developed in the future. In this study, we conducted 12 weeks of in vitro tissue integration experiments, however, long-term development and survival of such human chondrocyte-encapsulated implants beyond 12 weeks, as well as their responses in vivo, require further investigation. Overall, the findings derived from this work not only provide valuable information to surgeons who perform MACT for treating human cartilage degeneration in the future, but also open a new avenue towards improvement in designing hydrogel platforms suitable for clinical uses.

## Figures and Tables

**Table 1:** Physical properties of agarose hydrogels.

Agarose Hydrogels	Young’s Modulus ^1,^* (kPa)	Shear Modulus ^1,^* (kPa)	Water Content ^2,^* (%)	Mass Swelling Ratio ^2,^*
0.5%	0.49 ± 0.04	24.70 ± 1.75	99.60 ± 0.05	236.50 ± 2.30
1.0%	0.93 ± 0.04	40.93 ± 2.13	99.01 ± 0.17	94.00 ± 4.91
2.5%	3.30 ± 0.11	131.75 ± 3.96	97.78 ± 0.07	45.16 ± 1.46
5.0%	8.78 ± 0.78	344.88 ± 30.65	95.43 ± 0.12	21.88 ± 0.59
7.5%	14.60 ± 0.93	575.75 ± 37.21	93.46 ± 0.23	15.31 ± 0.53
10%	23.08 ± 1.31	902.28 ± 51.40	92.07 ± 0.18	8.73 ± 0.39

^1^ n = 14, ^2^ n = 5, and * *p* < 0.0001.

**Table 2:** Tissue properties of cartilage explants.

Thickness of Cartilage Discs (mm)	Equilibrium Modulus (kPa)	GAG (% of Wet Weight)	Total Collagen (% of Wet Weight)	Water Content (% of Wet Weight)
1.85 ± 0.39	871.24 ± 177.03	13.93 ± 2.37	20.59 ± 3.25	69.48 ± 0.97

n = 14 pooled together from three donors.

**Table 3:** Human donors used for extraction of articular chondrocytes and cartilage explants.

Donor ID #	Age	Gender	Cause of Death	Tissue Type	Extraction
0351504652	18	Female	Adverse effects of drugs	Osteochondral grafts	Articular chondrocytes
0211504897	20	Male	Multiple trauma		
0315004996	17	Male	Asthma attack		
0331505440	18	Male	Cardiac arrest		
0951604998	18	Male	Multiple trauma		
0671600155	41	Female	Cerebrovascular accident	Whole femur	Articular cartilage explants
0431602435	31	Male	Hanging		
0951603508	31	Male	Overdose		

## References

[1] Barbour K.E., Helmick C.G., Boring M., Brady T.J. (2017). Vital signs: Prevalence of doctor-diagnosed arthritis and arthritis-attributable activity limitation—United States, 2013–2015. Morb. Mortal. Wkly. Rep..

[2] Brown T.D., Johnston R.C., Saltzman C.L., Marsh J.L., Buckwalter J.A. (2006). Posttraumatic osteoarthritis: A first estimate of incidence, prevalence, and burden of disease. J. Orthop. Trauma.

[3] Yang Y.-H., Barabino G.A., Zhang L.G., Khademhosseini A., Webster T.J. (2014). Environmental factors in cartilage tissue engineering. Tissue and Organ Regeneration: Advances in Micro- and Nanotechnology.

[4] Kwon H., Brown W.E., Lee C.A., Wang D., Paschos N., Hu J.C., Athanasiou K.A. (2019). Surgical and tissue engineering strategies for articular cartilage and meniscus repair. Nat. Rev. Rheumatol..

[5] Johnstone B., Alini M., Cucchiarini M., Dodge G.R., Eglin D., Guilak F., Madry H., Mata A., Mauck R.L., Semino C.E. (2013). Tissue engineering for articular cartilage repair—The state of the art. Eur. Cells Mater..

[6] Basad E., Wissing F.R., Fehrenbach P., Rickert M., Steinmeyer J., Ishaque B. (2015). Matrix-induced autologous chondrocyte implantation (MACI) in the knee: Clinical outcomes and challenges. Knee Surg. Sports Traumatol. Arthrosc..

[7] Khan I.M., Gilbert S.J., Singhrao S.K., Duance V.C., Archer C.W. (2008). Cartilage integration: Evaluation of the reasons for failure of integration during cartilage repair. A review. Eur. Cells Mater..

[8] Gilbert S.J., Singhrao S.K., Khan I.M., Gonzalez L.G., Thomson B.M., Burdon D., Duance V.C., Archer C.W. (2009). Enhanced tissue integration during cartilage repair in vitro can be achieved by inhibiting chondrocyte death at the wound edge. Tissue Eng. Part A.

[9] Djouad F., Rackwitz L., Song Y., Janjanin S., Tuan R.S. (2009). ERK1/2 activation induced by inflammatory cytokines compromises effective host tissue integration of engineered cartilage. Tissue Eng. Part A.

[10] Mastbergen S.C., Saris D.B.F., Lafeber F.P.J.G. (2013). Functional articular cartilage repair: Here, near, or is the best approach not yet clear?. Nat. Rev. Rheumatol..

[11] Deponti D., Di Giancamillo A., Mangiavini L., Pozzi A., Fraschini G., Sosio C., Domeneghini C., Peretti G.M. (2012). Fibrin-based model for cartilage regeneration: Tissue maturation from in vitro to in vivo. Tissue Eng. Part A.

[12] Guilak F., Cohen D.M., Estes B.T., Gimble J.M., Liedtke W., Chen C.S. (2009). Control of stem cell fate by physical interactions with the extracellular matrix. Cell Stem Cell.

[13] Engler A.J., Sen S., Sweeney H.L., Discher D.E. (2006). Matrix elasticity directs stem cell lineage specification. Cell.

[14] Leipzig N.D., Shoichet M.S. (2009). The effect of substrate stiffness on adult neural stem cell behavior. Biomaterials.

[15] Smith Callahan L.A., Ganios A.M., Childers E.P., Weiner S.D., Becker M.L. (2013). Primary human chondrocyte extracellular matrix formation and phenotype maintenance using RGD-derivatized PEGDM hydrogels possessing a continuous Young’s modulus gradient. Acta Biomater..

[16] Subramanian A., Lin H.-Y. (2005). Crosslinked chitosan: Its physical properties and the effects of matrix stiffness on chondrocyte cell morphology and proliferation. J. Biomed. Mater. Res. A.

[17] Discher D.E., Janmey P., Wang Y.-L. (2005). Tissue cells feel and respond to the stiffness of their substrate. Science.

[18] Yang Y.-H., Ard M.B., Halper J.T., Barabino G.A. (2014). Type I collagen-based fibrous capsule enhances integration of tissue-engineered cartilage with native articular cartilage. Ann. Biomed. Eng..

[19] Mauck R.L., Soltz M.A., Wang C.C.B., Wong D.D., Chao P.-H.G., Valhmu W.B., Hung C.T., Ateshian G.A. (2000). Functional tissue engineering of articular cartilage through dynamic loading of chondrocyte-seeded agarose gels. J. Biomech. Eng..

[20] Yang Y.-H., Lee A.J., Barabino G.A. (2012). Coculture-driven mesenchymal stem cell-differentiated articular chondrocyte-like cells support neocartilage development. Stem Cells Transl. Med..

[21] Balgude A.P., Yu X., Szymanski A., Bellamkonda R.V. (2001). Agarose gel stiffness determines rate of DRG neurite extension in 3D cultures. Biomaterials.

[22] Walker J.M., Myers A.M., Schluchter M.D., Goldberg V.M., Caplan A.I., Berilla J.A., Mansour J.M., Welter J.F. (2011). Nondestructive evaluation of hydrogel mechanical properties using ultrasound. Ann. Biomed. Eng..

[23] Benya P.D., Shaffer J.D. (1982). Dedifferentiated chondrocytes reexpress the differentiated collagen phenotype when cultured in agarose gels. Cell.

[24] Benkherourou M., Rochas C., Tracqui P., Tranqui L., Gume’ry P.Y. (1999). Standardization of a method for characterizing low-concentration biogels: Elastic properties of low-concentration agarose gels. J. Biomech. Eng..

[25] Normand V., Lootens D.L., Amici E., Plucknett K.P., Aymard P. (2000). New insight into agarose gel mechanical properties. Biomacromolecules.

[26] Subhash G., Liu Q., Moore D.F., Ifju P.G., Haile M.A. (2011). Concentration dependence of tensile behavior in agarose gel using digital image correlation. Exp. Mech..

[27] Kazi G.A.S., Rahman K.A., Farahat M., Matsumoto T. (2019). Fabrication of single gel with different mechanical stiffness using three-dimensional mold. J. Biomed. Mater. Res. A.

[28] Beck E.C., Barragan M., Libeer T.B., Kieweg S.L., Converse G.L., Hopkins R.A., Berkland C.J., Detamore M.S. (2016). Chondroinduction from naturally derived cartilage matrix: A comparison between devitalized and decellularized cartilage encapsulated in hydrogel pastes. Tissue Eng. Part A.

[29] Ignotz R.A., Massagué J. (1986). Transforming growth factor-β stimulates the expression of fibronectin and collagen and their incorporation into the extracellular matrix. J. Biol. Chem..

[30] Childs C.B., Proper J.A., Tucker R.F., Moses H.L. (1982). Serum contains a platelet-derived transforming growth factor. Proc. Natl. Acad. Sci. USA.

[31] Bonner J.C. (2004). Regulation of PDGF and its receptors in fibrotic diseases. Cytokine Growth Factor Rev..

[32] Shapiro F., Koide S., Glimcher M.J. (1993). Cell origin and differentiation in the repair of full-thickness defects of articular cartilage. J. Bone Joint Surg. Am..

[33] Knutsen G., Engebretsen L., Ludvigsen T.C., Drogset J.O., Grøntvedt T., Solheim E., Strand T., Roberts S., Isaksen V., Johansen O. (2004). Autologous chondrocyte implantation compared with microfracture in the knee. J. Bone Jt. Surg. Am..

[34] Sharma B., Fermanian S., Gibson M., Unterman S., Herzka D.A., Cascio B., Coburn J., Hui A.Y., Marcus N., Gold G.E. (2013). Human cartilage repair with a photoreactive adhesive-hydrogel composite. Sci. Transl. Med..

[35] Vinardell T., Thorpe S., Buckley C., Kelly D. (2009). Chondrogenesis and integration of mesenchymal stem cells within an in vitro cartilage defect repair model. Ann. Biomed. Eng..

[36] Peretti G.M., Campo-Ruiz V., Gonzalez S., Randolph M.A., Wei X.J., Morse K.R., Roses R.E., Yaremchuk M.J. (2006). Tissue engineered cartilage integration to live and devitalized cartilage: A study by reflectance mode confocal microscopy and standard histology. Connect. Tissue Res..

[37] Darling E.M., Athanasiou K.A. (2005). Rapid phenotypic changes in passaged articular chondrocyte subpopulations. J. Orthop. Res..

[38] Murphy M.K., Huey D.J., Hu J.C., Athanasiou K.A. (2015). TGF-β1, GDF-5, and BMP-2 stimulation induces chondrogenesis in expanded human articular chondrocytes and marrow-derived stromal cells. Stem Cells.

[39] DiMicco M.A., Waters S.N., Akeson W.H., Sah R.L. (2002). Integrative articular cartilage repair: Dependence on developmental stage and collagen metabolism. Osteoarthr. Cartil..

[40] Hunter C.J., Levenston M.E. (2004). Maturation and integration of tissue-engineered cartilages within an in vitro defect repair model. Tissue Eng..

[41] Obradovic B., Martin I., Padera R.F., Treppo S., Freed L.E., Vunjak-Navakovic G. (2001). Integration of engineered cartilage. J. Orthop. Res..

[42] Tognana E., Chen F., Padera R.F., Leddy H.A., Christensen S.E., Guilak F., Vunjak-Novakovic G., Freed L.E. (2005). Adjacent tissues (cartilage, bone) affect the functional integration of engineered calf cartilage in vitro. Osteoarthr. Cartil..

[43] Mosley M.C., Lim H.J., Chen J., Yang Y.-H., Li S., Liu Y., Smith Callahan L.A. (2017). Neurite extension and neuronal differentiation of human induced pluripotent stem cell derived neural stem cells on a polyethylene glycol hydrogels containing a continuous Young’s modulus gradient. J. Biomed. Mater. Res. A.

[44] Lee J., Abdeen A.A., Zhang D., Kilian K.A. (2013). Directing stem cell fate on hydrogel substrates by controlling cell geometry, matrix mechanics and adhesion ligand composition. Biomaterials.

[45] Klotz B.J., Gawlitta D., Rosenberg A.J.W.P., Malda J., Melchels F.P.W. (2016). Gelatin-methacryloyl hydrogels: Towards biofabrication-based tissue repair. Trends Biotechnol..

[46] Antoine E.E., Vlachos P.P., Rylander M.N. (2014). Review of collagen I hydrogels for bioengineered tissue microenvironments: Characterization of mechanics, structure, and transport. Tissue Eng. Part B Rev..

[47] Merrill E.W., Dennison K.A., Sung C. (1993). Partitioning and diffusion of solutes in hydrogels of poly(ethylene oxide). Biomaterials.

[48] Cruise G.M., Scharp D.S., Hubbell J.A. (1998). Characterization of permeability and network structure of interfacially photopolymerized poly(ethylene glycol) diacrylate hydrogels. Biomaterials.

[49] Yang Y.-H., Khan Z., Ma C., Lim H.J., Smith Callahan L.A. (2015). Optimization of adhesive conditions for neural differentiation of murine embryonic stem cells using hydrogels functionalized with continuous Ile-Lys-Val-Ala-Val concentration gradients. Acta Biomater..

[50] Yang Y.-H., Barabino G.A. (2011). Requirement for serum in medium supplemented with insulin-transferrin-selenium for hydrodynamic cultivation of engineered cartilage. Tissue Eng. Part A.

[51] Yang Y.-H., Barabino G.A. (2013). Differential morphology and homogeneity of tissue-engineered cartilage in hydrodynamic cultivation with transient exposure to insulin-like growth factor-1 and transforming growth factor-β1. Tissue Eng. Part A.

[52] Kim Y.-J., Sah R.L.Y., Doong J.-Y.H., Grodzinsky A.J. (1988). Fluorometric assay of DNA in cartilage explants using Hoechst 33258. Anal. Biochem..

[53] Farndale R.W., Buttle D.J., Barrett A.J. (1986). Improved quantitation and discrimination of sulphated glycosaminoglycans by use of dimethylmethylene blue. Biochim. Biophys. Acta.

[54] Woessner J.F. (1961). The determination of hydroxyproline in tissue and protein samples containing small proportions of this imino acid. Arch. Biochem. Biophys..

[55] Tronci G., Grant C.A., Thomson N.H., Russell S.J., Wood D.J. (2015). Multi-scale mechanical characterization of highly swollen photo-activated collagen hydrogels. J. R. Soc. Interface.

[56] Peretti G.M., Zaporojan V., Spangenberg K.M., Randolph M.A., Fellers J., Bonassar L.J. (2003). Cell-based bonding of articular cartilage: An extended study. J. Biomed. Mater. Res. A.

[57] Yang Y.-H.K., Ogando C.R., Wang See C., Barabino G.A. (2017). Effect of hydrogel mechanics on in-situ cartilage integration. Osteoarthr. Cartil..

